# Informal and Formal Social Support and Caregiver Burden: The AGES Caregiver Survey

**DOI:** 10.2188/jea.JE20150263

**Published:** 2016-12-05

**Authors:** Koichiro Shiba, Naoki Kondo, Katsunori Kondo

**Affiliations:** 1 Departments of Health and Social Behavior/Health Education and Health Sociology, School of Public Health, The University of Tokyo, Tokyo, Japan; 2 Center for Preventive Medical Sciences, Chiba University, Chiba, Japan; 3 Department of Gerontological Evaluation, Center for Gerontology and Social Science, National Center for Geriatrics and Gerontology, Aichi, Japan

**Keywords:** social support, caregiver’s burden, long-term care, Japan, older people

## Abstract

**Background:**

We examined the associations of informal (eg, family members and friends) and formal (eg, physician and visiting nurses) social support with caregiver’s burden in long-term care and the relationship between the number of available sources of social support and caregiver burden.

**Methods:**

We conducted a mail-in survey in 2003 and used data of 2998 main caregivers of frail older adults in Aichi, Japan. We used a validated scale to assess caregiver burden.

**Results:**

Multiple linear regression demonstrated that, after controlling for caregivers’ sociodemographic and other characteristics, informal social support was significantly associated with lower caregiver burden (β = −1.59, *P* < 0.0001), while formal support was not (β = −0.30, *P* = 0.39). Evaluating the associations by specific sources of social support, informal social supports from the caregiver’s family living together (β = −0.71, *P* < 0.0001) and from relatives (β = −0.61, *P* = 0.001) were associated with lower caregiver burden, whereas formal social support was associated with lower caregiver burden only if it was from family physicians (β = −0.56, *P* = 0.001). Compared to caregivers without informal support, those who had one support (β = −1.62, *P* < 0.0001) and two or more supports (β = −1.55, *P* < 0.0001) had significantly lower burden. This association was not observed for formal support.

**Conclusions:**

Social support from intimate social relationships may positively affect caregivers’ psychological wellbeing independent of the receipt of formal social support, resulting in less burden.

## INTRODUCTION

Population aging is a global phenomenon. Many countries are implementing wide-ranging healthcare and long-term care (LTC) reforms to maintain sustainability in their financing and care provision systems. Reducing caregiver burden is particularly important, as caregiver burden hinders the health outcomes of disabled older adults and caregiver performance. Family caregivers experience a substantial sense of burden,^1^^–^^3^ resulting in psychiatric and physical illnesses^4^^–^^7^ and mortality.^8^

Japan is the leading country in its pace of population aging. In 2012, there were approximately 5.3 million adults eligible for the public LTC insurance benefit and thus potentially requiring LTC.^9^ Reducing the burden of family caregivers is especially important in Japan because of the strong social norm of caring for all family members within the family, stemming from Japanese traditional values regarding family conception.^3^^,^^10^ A government survey reported that approximately 60% of family caregivers cohabitating with older people in their care felt worries and stress.^11^

Social support for caregivers is a key target of interventions to reduce caregiver burden.^12^^–^^17^ To date, studies have revealed that emotional, instrumental, appraisal, and informational supports for caregivers may reduce caregiver burden.^18^^,^^19^ Although these supports could be provided through caregivers’ formal and informal social relationships, epidemiologic studies have mainly focused on social support gained from caregiver’s *informal* relationships, such as family members, friends, and neighbors. *Formal* social support from professionals/public services (eg, family physicians, nurses, and social workers) may also be effective in reducing caregiver burden. However, few studies have simultaneously examined the effects of formal and informal social supports on reducing caregiver burden. Moreover, the effects of social support on caregiver burden may differ by its sources.^20^^,^^21^ For example, family members’ support may be specifically effective as emotional support, and the number of available sources of support may be important. Hence, we hypothesized that having more sources of social support may be advantageous for caregivers.

Accordingly, we sought to examine (1) the independent association of either informal or formal social support with caregiver burden, (2) the relationship between social support from each source and caregiver burden, and (3) the relationship between the number of available sources of social support and caregiver burden.

## METHODS

### Data

Data were derived from the Caregiver Survey under the Aichi Gerontological Evaluation Study (AGES) project.^22^ A postal survey was mailed to all caregivers of physically and/or cognitively impaired individuals certified as being eligible for public LTC insurance benefits and using in-home services covered by seven insurers (municipality governments) of Aichi Prefecture, Japan, in May 2003. Among 3610 subjects who responded to the survey (response rate: 50%), data obtained from primary caregivers, who were mainly in charge of caregiving, were used (*n* = 3149). Respondents with missing values for sex, age, and our caregiver burden scale were excluded; ultimately, 2998 individual observations were used in the analysis.

### Measurements

#### Caregiver burden

Caregiver burden was measured using a validated 8-item scale, the revised edition of Nihon Fukushi University Caregiver Burden Scale.^23^^,^^24^ This scale consists of three subscales: ‘subjective caregiver burden’, ‘intention to continue caregiving’, and ‘social norms toward caregiving’. We used subjective caregiver burden as the outcome in this study. The caregiver burden score was calculated by summing the score of each answer to the eight questions (possible score ranges from 1 to 4 per question), with total caregiver burden scores ranging from 8 to 32; higher score reflect higher burden. Following the suggested imputation method for missing values in a scale variable,^25^ we used the mean value of the rest of the answers when there was a missing value in only one question out of eight. When a respondent had two or more missing values, we treated the caregiver burden score as missing (*n* = 95).

#### Social support

Social support was measured by asking, ‘Do you have anyone to consult when you have trouble with caregiving?’ From the lists of potential sources of support, respondents were asked to select all the sources of informal/formal social support they had. We defined informal social support as support from the caregiver’s family living together, children living apart, relatives, friends, neighbors, and other non-professionals. We defined formal social support as support from the caregiver’s family physicians, care managers (registered professionals who plan and manage LTC schedules for older persons with disability), home-helpers, visiting nurses, public health nurses, social workers, officers in public institutions, and other professionals. The number of available sources of social support was categorized as 0, 1, and ≥2. It should be noted that the obtained information reflects caregiver’s subjective perception of social support and may differ from actual receipt of social support.

#### Covariates

Age and gender of respondents, level of necessary LTC, household income, cohabitation with care recipients, duration of caregiving, average daily caregiving time, relationships with care recipients, dementia severity of care recipients, a sense of hesitation regarding use of public caregiving services, use of formal in-home care services, and existence of sub-caregivers were treated as covariates.

We used government-certified levels for necessary LTC. The seven levels for necessary LTC (Support level-1, Support level-2, LTC-1, LTC-2, LTC-3, LTC-4, and LTC-5) were determined using the criteria developed by the Ministry of Health, Labour and Welfare (MHLW).^9^ Support levels-1 and -2 are defined as conditions requiring less care that can improve or in which activities of daily living (ADLs) can be maintained with proper care. LTC-1 and -2 refer to more severe conditions that require only partial support to accomplish basic ADLs (eg, toileting and bathing). People at LTC-3 or greater are completely dependent on assistance for many ADLs, ranging from toileting and bathing to rising, dressing, and even communicating.^26^ As such, we categorized subjects as ‘Support level-1 or -2’ (least severe), ‘LTC-1 or -2’, or ‘LTC-3 or greater’ (most severe).

Dementia severity was also assessed based on criteria developed by MHLW.^27^ We categorized subjects as ‘no dementia’, ‘Rank I’, and ‘Rank II or more’. Patients with Rank II or more have difficulty with independent daily living and require care and support, while Rank I patients can live independently.

We assessed sense of hesitation regarding use of public caregiving services, asking caregivers to rate the degree to which they hesitate over using public services for caregiving due to social pressures from relatives and neighbors. Subjects were then categorized as either feeling or not feeling hesitation.

Household income was equivalized in order to adjust for the number of household members. Equivalized household income, duration of caregiving, and average daily caregiving time were categorized into tertiles.

Respondents were asked to select all the formal in-home care services they were using from a list of potential services. Caregivers using at least one of these services were categorized as “using services”.

Existence of sub-caregivers was measured by the dichotomous question: “Do you have anyone who can substitute for you in caregiving (except professional workers in caregiving, such as home helpers)?”.

### Statistical analysis

We used multiple linear regression models to examine the impact of informal/formal social support on caregiver burden scores. We conducted three different analyses. For each analysis, we used (1) the existence of perceived social support (no perceived social support vs one or more perceived social support), (2) perceived social support from each source, and (3) the number of available sources of social support (0, 1, or ≥2) as explanatory variables. We conducted trend tests using a discrete variable of the number of available sources of social support (0 = no support, 1 = one support, and 2 = two or more supports). In the models, caregiver’s burden score and caregiver’s age were treated as continuous variables. Other variables were treated as categorical variables and were modelled using dummy variables (including missing dummy variables). SAS 9.3 (SAS Institute, Cary, NC, USA) was used for all analyses.

## RESULTS

Descriptive statistics showed that caregivers who were female, earning more income, caring for a longer duration, and having a sub-caregiver were more likely to report having at least one informal social support. Caregivers who were not cohabitating with care recipients, who had longer average daily caregiving time, and who had a sub-caregiver were more likely to report having at least one formal social support (Table 1).

**Table 1.** Proportions of having informal and formal social support by demographic characteristics of caregivers

		Informal social support^b^			Formal social support^c^		
	
		*n*	%	*P*-value^a^	*N*	%	*P*-value^a^
Total		2775	92.6		2814	93.9	
Gender	Male	596	90.0	<0.0001	623	93.0	0.28
	Female	2179	93.6		2191	94.1	
Levels for necessary LTC	Support level-1, -2	247	94.3	0.49	242	92.4	0.23
	LTC-1, -2	1448	92.2		1470	93.6	
	LTC-3 or greater	1017	92.5		1042	94.8	
	Missing	63			60		
Age, years	<60	1362	93.4	0.24	1367	93.7	0.11
	60–75	1036	91.6		1072	94.8	
	≥75	377	92.4		375	91.9	
Household income, 10 000 Japanese yen	<300	757	89.4	<0.0001	799	94.3	0.94
	300–600	841	93.3		849	94.2	
	≥600	931	95.2		925	94.6	
	Missing	246			241		
Cohabitation with care recipients	+	2401	92.6	0.93	2422	93.4	0.003
	−	367	92.4		386	97.2	
	Missing	7			6		
Average daily caregiving time, hours	<2	511	94.11	0.24	509	93.7	0.48
	2–5	996	92.57		1020	94.8	
	≥5	876	91.73		909	95.2	
	Missing	392			376		
Duration of caregiving, months	<30	739	91.9	0.06	754	93.8	0.66
	30–60	1024	94.0		1020	93.7	
	≥60	891	91.5		921	94.6	
	Missing	121			119		
Relationships with care recipients	Spouse	796	90.9	0.002	817	93.3	0.89
	Daughter-in-law	982	94.9		977	94.4	
	Daughter	629	93.2		636	94.2	
	Son	264	89.8		276	93.9	
	Sibling	31	91.2		31	91.2	
	Others	64	86.5		69	93.2	
	Missing	9			8		
Severity of dementia	No dementia	1091	92.7	0.93	1104	93.8	0.29
	I	581	92.5		583	92.8	
	II or more	1040	92.3		1067	94.7	
	Missing	63			60		
Have a sense of hesitation regarding use of public caregiving services	Yes	378	90.7	0.10	401	91.3	0.11
	No	2386	92.9		2450	93.9	
	Missing	11			13		
Use of formal in-home care services	Yes	2727	92.6	0.30	2764	93.9	0.69
	No	48	88.9		50	92.6	
Existence of sub-caregivers	Yes	1155	96.8	<0.0001	1142	95.7	0.001
	No	1552	89.6		1608	92.8	
	Missing	68			64		

LTC, long-term care.

^a^Chi-square test by excluding missing values.

^b^Informal social support: social support from caregiver’s family living together, children living apart, relatives, friends, neighbors, and others.

^c^Formal social support: social from caregiver’s family doctors, care managers, home-helpers, visiting nurses, public health nurses, social workers, officers in public institutions, and others.

Multiple linear regression showed that, after adjusting for covariates listed in Table 2, caregiver burden score was 1.59 points lower among those with at least one informal social support compared to those without informal social support (*P* < 0.0001). Conversely, we did not observe a significant association between formal social support and caregiver burden (β = −0.30, *P* = 0.39; Table 2).

**Table 2.** Linear regression model for caregiver’s burden score by having informal and/or formal social support (*n* = 2998)

Independent variable	Crude		Model 1 (Adjusted)	

	β Coefficient	*P*-value	β Coefficient	*P*-value
Social support				
Informal social support^a^	−2.03	<0.0001	−1.59	<0.0001
Formal social support^b^	−0.77	0.04	−0.30	0.39
Gender				
Male	0	Reference	0	Reference
Female	0.64	0.003	1.06	00.0001
Age	0.03	<0.0001	0.02	0.07
Levels for necessary LTC				
Support level-1, -2	0	Reference	0	Reference
LTC-1, -2	1.09	<0.0001	0.32	0.28
LTC-3 or more	3.51	<0.0001	1.70	<0.0001
Equivalized household income, 10 000 yen^c^				
Low	0	Reference	0	Reference
Middle	−0.14	0.52	−0.11	0.56
High	−0.47	0.49	−0.04	0.95
Cohabitation with care recipients				
No	0	Reference	0	Reference
Yes	1.79	<0.0001	0.32	0.22
Duration of caregiving^c^				
Short	0	Reference	0	Reference
Middle	−0.04	0.85	0.06	0.76
Long	0.58	0.03	0.33	0.16
Average daily caregiving time^c^				
Short	0	Reference	0	Reference
Middle	1.26	<0.0001	0.82	<0.0001
Long	1.89	<0.0001	1.08	0.0002
Severity of dementia				
No dementia	0	Reference	0	Reference
I	1.16	<0.0001	1.02	<0.0001
II or more	3.32	<0.0001	2.50	<0.0001
Relationships with care recipients				
Spouse	0	Reference	0	Reference
Daughter-in-law	−0.43	0.05	−0.46	0.08
Daughter	−1.24	<0.0001	−0.97	0.00
Son	−1.41	<0.0001	0.13	0.71
Sibling	−0.62	0.47	−0.48	0.55
Others	−1.92	0.00	−1.32	0.02
A sense of hesitation regarding use of public caregiving services				
No	0	Reference	0	Reference
Yes	3.30	<0.0001	2.96	<0.0001
Use of formal in-home care services				
No	0	Reference	0	Reference
Yes	1.18	0.08	0.77	0.20
Existence of sub-caregivers				
No	0	Reference	0	Reference
Yes	−1.34	<0.0001	−0.97	<0.0001

LTC, long-term care.

Caregiver’s burden score ranged from 8 to 32 points.

^a^Informal social support: social support from caregiver’s family living together, children living apart, relatives, friends, neighbors, and others.

^b^Formal social support: social support as the social support from caregiver’s family doctors, care managers, home-helpers, visiting nurses, public health nurses, social workers, officers in public institutions, and others.

^c^Equivalized household income, duration of caregiving, average daily caregiving time were categorized using tertiles.

When evaluating the associations by specific sources of informal/formal social supports, informal social supports from caregiver’s family living together (β = −0.71, *P* < 0.0001) and relatives (β = −0.61, *P* = 0.001), and formal social support from family physicians (β = −0.56, *P* = 0.001) had significant associations with lower caregiver burden. Contrarily, caregiver burden score was 0.52 points higher among caregivers with formal social support from care managers compared to those without such support (*P* = 0.01; Model 2 in Table 3).

**Table 3.** Multiple linear regression model for caregiver’s burden score by specific sources of informal and/or formal social support (Model 2) and by the number of informal and/or formal social supports (Model 3) (*n* = 2998)

	Model 2		Model 3	

Independent variable	β Coefficient	*P*-value	β Coefficient	*P*-value
Informal social support				
Caregiver’s family living together	−0.71	<0.0001		
Children living apart	0.09	0.61		
Relatives	−0.61	0.001		
Friends	−0.02	0.93		
Neighbors	0.06	0.82		
Others	−0.24	0.60		
Formal social support				
Caregiver’s family physicians	−0.56	0.001		
Care managers	0.52	0.01		
Home-helpers	0.02	0.93		
Visiting nurses	0.33	0.25		
Public health nurses	0.86	0.17		
Social workers	0.67	0.30		
Officers in public institutions	0.001	1.00		
Others	0.44	0.52		

Informal social support				
None			0	Reference
1			−1.62	<0.0001
≥2			−1.55	<0.0001
*P* for trend			0.003	
Formal social support				
0			0	Reference
1			−0.30	0.39
≥2			−0.32	0.38
*P* for trend			0.92	

All variables regarding social support in each model were modelled simultaneously.

The model was adjusted for age and gender of respondents, the level of necessary long-term care, equivalized household income, cohabitation with care recipients, duration of caregiving, average daily caregiving time, relationships with care recipients, the severity of dementia of care recipients, a sense of hesitation regarding use of public caregiving services, use of formal in-home care services, and existence of sub-caregivers.

In the regression model with the number of available sources of social support, caregiver burden score was 1.62 points lower among those with one informal social support (*P* < 0.0001) and 1.55 points lower among those with two or more informal social supports (*P* < 0.0001) compared to caregivers without any informal social support. Trend tests showed a significant trend for informal social support (*P* = 0.003). This was not observed for formal social support (*P* = 0.92; Model 3 in Table 3).

## DISCUSSION

To our knowledge, this was the first study to evaluate the association of informal and formal social support with caregiver’s burden using detailed information on the number and sources of social support. The key findings of this study are threefold. First, having informal social support was associated with lower caregiver burden, while formal social support was not. Second, significantly lower caregiver burden was observed among caregivers with informal social support from caregiver’s family living together and relatives, while, among the sources of formal social support, only support from family physicians was significantly associated with lower caregiver burden. Finally, we did not find an association between the number of available sources of social support and level of caregiver burden.

Our findings are consistent with preceding studies on the association between receipt of informal social support and reduced caregiver burden.^12^^,^^16^^,^^17^ Our study adds a new finding: the beneficial effect of informal social support may be independent of whether caregivers have formal social support. Our finding showing less support among those with low income is also consistent with recent studies in the United States.^20^^,^^21^

Notably, we found that informal social support from intimate family/relatives may be specifically beneficial, independent of the receipt of formal social support. Although we did not evaluate types of social support, we speculate that intimate family members may be helpful specifically in terms of emotional social support. Second, regarding informal social support, perception of at least one form of informal social support was associated with lower caregiver burden. In particular, perception of social support from the caregiver’s family living together and children living apart were significantly associated with lower burden. Previous studies have also argued that social support from caregivers’ informal interpersonal relationships may attenuate caregiver burden.^12^^,^^16^^,^^17^ Thoits^20^ and Lin et al^28^ argued that emotional social support has a more direct and positive influence on psychological wellbeing than informational and instrumental support. This may also explain the overall non-significant results for formal social support. In other words, caregivers may mainly rely on professional supporters, seeking information about public services in caregiving and instrumental support (eg, actual nursing care and domestic assistance) but not expecting emotional support.

Formal social support from family physicians was associated with lower burden, independent of the receipt of informal social support. This may imply that family physicians play an important role in reducing caregiver burden when they are without informal social support. Although convincing evidence is lacking, caregivers may be more likely to have a sense of trust, reliance, and respect for family physicians—professionals with high social status—which may lead to a sense of relief when meeting family physicians. Further, family physicians may meet a caregiver more frequently than nurses, since family physicians usually care exclusively for patients, resulting in provision of more emotional support than other professionals, whereas visiting nurses can change with each visit.

We did not observe differences in caregiver burden by the number of available sources of informal social support, despite a significant *P*-value for the trend test. Findings from some studies support our result. White et al^29^ found that the extent to which social support is perceived to be helpful was a better predictor of psychological wellbeing than the number of available sources of social support. It may be that we did not find a ‘dose-response' relationship because informal social support from a caregiver’s casual social relationships was not as effective as support from more meaningful relationships, such as the caregiver’s family, so they did not have an impact on caregiver burden.

We must exercise caution to avoid interpreting our findings to suggest that formal support does not matter. Providing formal social support is essential to allow leisure time for caregivers. Without leisure time, it would be impossible for caregivers to seek sufficient informal social support, which requires social interactions with their intimate family members and friends.

Several limitations should be noted. First, this study was conducted with cross-sectional data. Therefore, reverse causation, in which caregivers seek and perceive social support when they feel greater burden, is possible. This may explain the association between perceived social support from care managers and increased caregiver burden observed in the present study. Even though we controlled for several possible confounding factors, future studies should use longitudinal data to permit causal inference. Second, the generalizability of the results is limited. The sample was collected from a single prefecture of Japan. As such, our sample may not be nationally representative of Japanese older individuals requiring care. Third, we did not specify the type of social support caregivers perceive. Uniting these different types of social support as a single variable may end up attenuating the statistical significance of the effect of social support. Identifying whether emotional, informational, or instrumental support is implicated in perceived social support would lead to a more profound understanding of the effect of social support in reducing caregiver burden. Fourth, the response rate of 50% was relatively low. Although the demographic and medical backgrounds of non-respondents of this survey are unknown, a preceding study using another dataset of the AGES project indicated that response rates among the lower income categories were lower than higher income categories.^22^ If this is also the case for the data we used in this study, socially vulnerable and possibly unhealthy caregivers would be more likely to be non-respondents. Therefore, our findings may underestimate the true effect.

### Conclusion

Our study has important public health implications. To reduce caregiver burden, increasing social support from caregivers’ intimate relationships and family physicians is important. In line with the present findings, informal social support is required regardless of the availability of formal social support. As our descriptive data showed that socioeconomically disadvantaged caregivers are more likely not to have any social support, they should be prioritized for targeted interventions.
